# Glaucoma surgery during the first year of the COVID-19 pandemic

**DOI:** 10.1007/s10792-022-02278-6

**Published:** 2022-04-16

**Authors:** Rosa Longo, Elia Franzolin, Emilio Pedrotti, Adriano Fasolo, Erika Bonacci, Giorgio Marchini

**Affiliations:** Department of Neurosciences, Biomedicine and Movement Sciences, Ophthalmic Unit, University of Verona, P.le L. A. Scuro 10, 37134 Verona, Italy

**Keywords:** COVID-19 pandemic, Glaucoma, Surgery, Eye

## Abstract

**Purpose:**

To summarize the actions taken to give continuity to the surgical treatment of glaucoma patients and to present the volume and characteristics of glaucoma surgery in the first year of pandemic at the Tertiary Glaucoma Center of the University Hospital of Verona (Veneto, Italy).

**Methods:**

Demographical and surgical features of patients who underwent glaucoma surgery from March 9th, 2020 to March 8th, 2021 have been collected and compared to the same date range of the previous year. The analyzed data included age, gender, region of origin, glaucoma staging, type of anesthesia and surgical procedure.

**Results:**

The surgical volume of glaucoma has dropped by 30.1%. In comparison with the previous year, we found a significant variation in the overall distribution of the performed surgical procedures (*p* < 0.001). There was a decline in Baerveldt tube implants (− 4.9%), and an increase of non-penetrating surgery (+ 2.6%), cyclo-photo ablative procedures (+ 4.2%) and MIGS (+ 5.7%). Only 24.3% of the procedures were performed under general anesthesia compared to 41.5% in the pre-pandemic period (*p* < 0.001). The number of procedures performed on eyes affected by advanced or end-stage glaucoma is doubled (*p* < 0.001).

**Conclusions:**

To give continuity to glaucoma surgery, we prioritized interventions on patients with poorer visual fields, rapidly progressing visual field deficit and elevated IOP uncontrolled by maximal medical therapy. Secondly, we have rescheduled the other interventions following the same priority criteria. Finally, we managed some lower priority cases with MIGS, minimizing the need for close post-intervention follow-up. Considering the negative consequences that a delay in the management of glaucoma can have in terms of visual loss, the closure of the operating rooms in the first quarter of the pandemic was detrimental. It appears that glaucoma surgery deserves urgencies that cannot be overshadowed and the greatest effort must be to give continuity to this type of eye surgery.

**Supplementary Information:**

The online version contains supplementary material available at 10.1007/s10792-022-02278-6.

## Introduction

The COVID-19 pandemic has caused an unprecedented overload of the National Health System in Italy. The spread of the contagion can be divided into three consecutive periods: the “first wave”, from March 9th 2020, began in the North of the country and was characterized by an exponential increase in the number of hospitalizations and deaths [1]. From June to August 2020, during what we identify as the “transition phase”, the diffusion was very limited. Since the end of August, several outbreaks had been identified throughout the country, resulting in a slow rise of daily infections. By October, the spread of the virus had already exceeded the levels reached in the spring, and the peak of the so-called second wave was in November*.* Thanks to the containment measures, weighed on the different regional scenarios, the infection was limited, without ever returning to the levels reached in the summer [2].

To face the high rate of admissions due to COVID-19, especially during the “first wave”, one of the measures adopted by the National Health System was to suspend elective surgical activities in favor of emergencies [3]. Therefore, many hospitals were intended exclusively for the management of COVID-19 patients, thus determining the withdrawal and rescheduling of elective surgical procedures [4]. These measures particularly interested the ophthalmic field, whose staff was at high risk of contracting the infection due to the close distance between clinician and patient during examination [5–7].

This study aims to summarize the actions taken to give continuity to the surgical treatment of glaucoma patients and to present the volume and characteristics of glaucoma surgery in the first year of the pandemic at the Tertiary Glaucoma Center of the University Hospital of Verona (Veneto, Italy). In March 2021, one year after the beginning of the pandemic, the Veneto region resulted among those most affected by the virus and 19% of confirmed regional cases were detected in Verona [8]. For these reasons, our geographical area can be representative of the COVID-19 emergency in Italy, and our eye center has been severely interested in the health crisis.

## Methods

This retrospective study was conducted at the Ophthalmology Clinic of the University Hospital of Verona, Italy. Demographical and surgical features of patients who underwent glaucoma surgery from March 9th, 2020 to March 8th, 2021 have been collected and compared to the same date range of the previous three years (2017, 2018 and 2019). Since the samples gathered in 2017, 2018 and 2019 did not differ significantly from each other for any of the collected variables (see supplementary material), only data relating to 2019 were considered for the statistical analysis.

The analyzed data included age, gender, region of origin, glaucoma staging based on the Glaucoma Staging System 2 [9], type of anesthesia and surgical procedure. Our study was approved by the Ethics Committee of Verona and Rovigo. All data were collected anonymously and according to the tenets of the Declaration of Helsinki. The informed consent for any surgical treatment and data processing was given by patients or by parents/authorized legal guardians for minors at the admission to the eye clinic.

Patients were phone-called one week before surgery and screened for COVID-19-related symptoms and risky contacts. As customary, in preparation for the surgical procedure, patients underwent routine blood tests and electrocardiograms. In addition, chest X-rays were performed for surgeries that required general anesthesia. A nose-pharyngeal swab for Sars-CoV-2 was executed 48 h before the surgery. If the nose-pharyngeal swab tested positive, the surgery had to be postponed until the negativization, unless the patient was at risk of irreversible and rapid vision loss. Interventions of infected patients were planned at the end of the operating list, with the operating room being be sanitized at the end of the procedure.

On the day of surgery, the body temperature of patients was assessed with a non-contact digital thermometer both when admitted to hospital and in the anteroom of the operating room. A maximum of one accompanying person per patient was allowed and patients were welcomed every 45 min to minimize crowds in the waiting rooms. Each patient sanitized hands upon entering the hospital and in the waiting room. At the operating room filters, patients sanitized their hands again, wore a surgical mask, hair cap, overshoes and disposable gown.

With exception of the dates from June 9th to September 8th 2020, we have experienced half the number of available operating rooms and the reduction in the nursing and anesthesia staff, who were recruited in COVID-19 areas.

Since the beginning of the pandemic, traveling between different geographical areas due to health reasons had always been allowed.

Descriptive results were reported as percentage for categorical variables and as mean ± Standard Deviation (SD) for quantitative ones. Chi-Square test and T test for independent means were used, respectively, to compare categorical and quantitative variables between the two periods. The analysis was done using STATA 16.0 (StataCorp, Texas, USA). Statistical significance was considered when *p* < 0.01.

## Results

Between March 9th, 2020 and March 8th, 2021, 490 surgeries were performed on 389 glaucomatous patients, compared with 701 surgeries on 494 patients carried out during the previous year. Therefore, the surgical volume of glaucoma has dropped by 30.1% and represented the 10.0 and the 8.4% of the total surgical ophthalmic activity during the pre-COVID period and COVID period, respectively.

Table 1 shows the patient’s demographics and surgical procedures carried out during the pandemic period and the corresponding time interval in the previous year.

**Table 1:** Demographics of patients and glaucoma surgical procedures performed during the first year of the COVID-19 pandemic

	Before pandemic (9th March 2019–8th March 2020)	During pandemic (9th March 2020–8th March 2021)	Variation (%)	*p*
*Demographics*				
Surgical procedures, *n*	701	490	− 30.1	**0.003**
Patients, *n*	494	389	− 21.3	0.813
Males, *n* (%)	258 (52.2)	208 (53.5)	+ 1.3	0.713
Age, mean (SD)	58.4 (23.4)	60.5 (21.4)	–	0.161
Advanced or End-Stage Glaucoma, *n* (%)	20 (4.1)	37 (9.4)	+ 5.3	** < 0.001**
Pediatric glaucoma, *n* (%)	69 (14.0)	28 (7.3)	+ 6.7	** < 0.001**
General anesthesia, *n* (%)	227 (32.4)	95 (19.4)	− 13.0	** < 0.001**
*Procedures, n (%)*				** < 0.001**
Examination under anesthesia	93 (13.3)	36 (7.3)	− 6.0	**0.001**
Trabeculotomy	6 (0.9)	10 (2.0)	+ 1.1	0.080
Trabeculectomy	119 (17.0)	63 (12.9)	− 4.1	0.041
Deep sclerectomy	66 (9.4)	59 (12.0)	+ 2.6	0.124
MIGS	22 (3.1)	43 (8.8)	+ 5.7	** < 0.001**
Baerveldt tube shunt	87 (12.4)	37 (7.6)	− 4.8	**0.007**
Baerveldt revision	90 (12.8)	44 (9.0)	− 3.8	0.038
Bleb revision	47 (6.7)	42 (8.6)	− 1.9	0.228
Transcleral cyclophotocoagulation	35 (5.0)	45 (9.2)	+ 4.2	**0.004**
Phaco for narrow-angle or PEX syndrome	121 (17.3)	104 (21.2)	− 3.9	0.086
Others*	15 (2.1)	7 (1.4)	− 0.7	0.370

*Scleral patch, iridoplastics, iridectomy, other interventions on the anterior chamber

MIGS: Minimally invasive glaucoma surgery; PEX: Pseudoexfoliation syndrome

Patients’ geographic provenience did not differ from those of the pre-pandemic period (Table 2 -supplementary material).

**Fig. 1:**
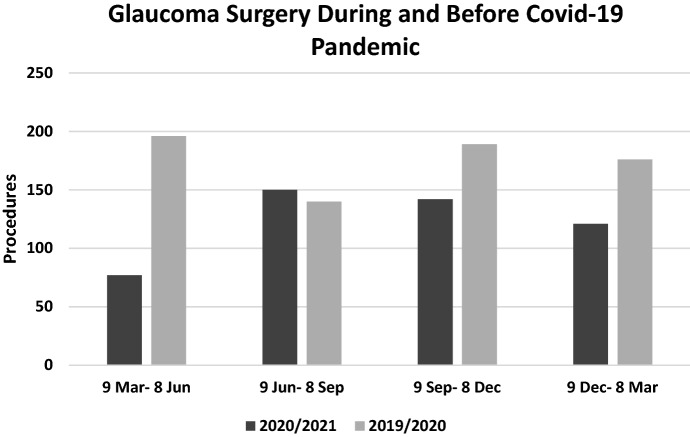
Number of glaucoma surgeries during and before the COVID-19 pandemic

Figure 1 shows the variation in the number of surgical procedures performed during the pandemic compared with those of the previous year. Dividing the pandemic period into quarters and comparing them with the corresponding date intervals in the previous year, we found that the greatest reduction in surgical activity, which reached − 60.7%, occurred during the first three months (March 9th–June 8th 2020). The second quarter (June 9th–September 8th 2020) was characterized by an increase of + 7.14%. A drop of − 24.9 and − 31.3% occurred, respectively, in the third quarter (September 9th–December 8th 2020) and in the fourth one (December 9th 2020–March 8th 2021).

During the pandemic, the number of procedures performed on eyes affected by advanced or end-stage glaucoma is doubled (*p* < 0.001). In addition, there was a halving of the infantile glaucomas (*p* < 0.001). In comparison with the previous year, we found a significant variation in the overall distribution of the performed surgical procedures (*p* < 0.001). There was a decline in the implantation of Baerveldt tubes (− 4.9%) and an increase in non-penetrating surgery (+ 2.6%), cyclo-photo ablative procedures (+ 4.2%) and MIGS (Minimally Invasive Glaucoma Surgery) (+ 5.7%).

Only 24.3% of the procedures were performed under general anesthesia compared to 41.5% in the pre-pandemic period (*p* < 0.001).

The rate of hospitalizations following glaucoma surgery during the pandemic was 19.5% (76 out of 389 patients), compared to 38.5% (190 out of 494 patients) in the pre-pandemic period.

Glaucoma surgeons and operating room staff periodically underwent both nose-pharyngeal swab or serology test and none of them resulted positive to COVID-19. Only one surgical candidate tested positive for preoperative swab and his surgery was postponed until negativization, being not at risk of rapid vision loss.

## Discussion

The aim of this study was to explain the measures adopted during the first year of the COVID-19 pandemic to give continuity to the glaucoma surgical care at the Ophthalmic Unit of the Tertiary University Hospital of Verona (Veneto, Italy) and to summarize the surgical activity performed despite the health emergency.

**Fig. 2:**
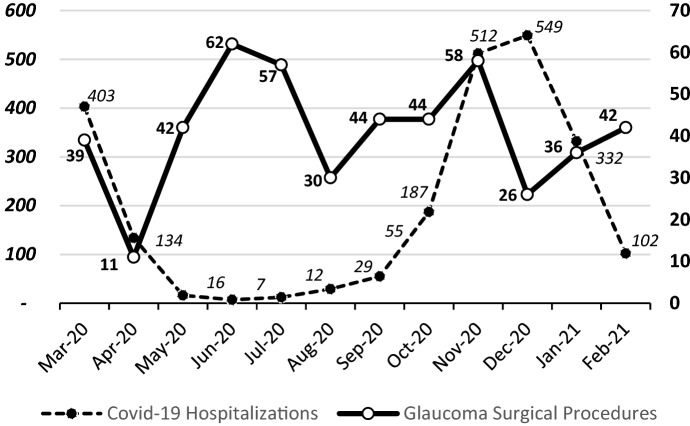
Number of monthly glaucoma surgical procedures in relation to hospitalizations for COVID-19 during the first year of COVID-19 Pandemic

Overall, during the first year of the pandemic, the volume of glaucoma surgery has dropped by 30.1%. The number of interventions performed in the four considered quarters was heavily influenced by hospitalizations and deaths caused by COVID-19 infection in Italy, as shown in Fig. 2.

The decrease in surgical activity observed in the first quarter, which temporally coincides with the national lockdown, was very serious. To the sudden interruption of elective surgeries imposed by Government directives, we have reacted in three different ways.

First, we prioritized interventions on patients with poorer visual fields, rapidly progressing visual field deficit, and elevated IOP uncontrolled by maximal medical therapy. Therefore, the surgical assistance continued, giving priority to those subjects at risk of faster glaucoma progression.

Secondly, we have rescheduled the other interventions following the same priority criteria.

The last action taken was to manage some lower priority cases with MIGS, minimizing the need for close post-intervention follow-up. These devices require less postoperative care and allow a temporary pressure reduction without excluding a possible future filtering intervention [5].

Due to the shortage of anesthesia staff, the most frequently postponed procedures have been the Baerveldt tube implants and diagnostic examinations under anesthesia. Procedures on children were mostly postponed as they require sedation or general anesthesia in most cases. As a consequence, trabeculectomies, deep sclerectomies and cyclo-photoablations, which required local anesthesia, have increased. Moreover, Baerveldt tube implants are more associated to postoperative complications than other surgeries and patients are usually hospitalized for one or a few days after surgery. Conversely, non-penetrating surgery requires fewer surgical revisions and is less associated with complications [10–12].

Compared to 41.5% of the pre-pandemic period, only 24.3% of the procedures were performed under general anesthesia, which therefore required the presence of the anesthetist in the operating room and subsequent hospitalization for one night. After reducing the need of anesthesist’ interventions by half, the positive consequences have been: (1) more availability of anesthesists to manage patients hospitalized in COVID-19 areas; (2) reduced need for admission of ophthalmic patients, in favor of one-day surgeries.

The increase in surgical activity that took place in the second quarter of the pandemic was significant, exceeding the number of operations carried out during the same date range of the previous year. This finding, which temporally coincided with the reduction in COVID-19 hospitalizations in Italy, was due to the restoration of the ordinary availability of operating theatres, nurses, anesthesia staff, and beds for patients undergoing ophthalmic surgery.

Despite this recovery, the decline suffered in the first quarter was never fully overcome during the year and some rescheduled patients underwent surgery late. This delay can partially be the cause of the doubling of cases of advanced and end-stage glaucoma recorded during the pandemic period. Another factor that may have influenced the increase in the number of advanced glaucoma was the closure of other glaucoma centers, which have been converted into COVID hospitals, so the most serious cases have reached our third level clinic.

Consistent with this finding, an increase in cyclo-photo ablative procedures occurred.

Even during the pandemic, patients who underwent glaucoma surgery continued to be checked regularly for 3 months by our medical staff. None of them showed symptoms of the COVID-19 infection or reported risky contacts. However, the nose-pharyngeal swab was no longer repeated after surgery, so asymptomatic infections can’t be excluded.

Our data are different from those reported by another third level Italian center [13], which reported a slight increase in the number of glaucoma surgeries performed during the first month of the pandemic. According to this center, the rise was consequent to the closure of smaller and suburban hospitals in their region. In the Veneto region, on the other hand, continued assistance was guaranteed by the ophthalmology centers.

The main weakness of this research is its retrospective and monocentric design, conversely the geographical position of our center, intensely affected by the health emergency makes it particularly representative in the national scenario. Furthermore, our clinic is a highly specialized third-level reference center for glaucoma, and this allowed us to treat a considerable number of patients.

## Conclusion

The goal of this work was to understand the impact of the measures adopted in the management of patients requiring glaucoma surgery in the first year of the pandemic in a third-level Italian center highly specialized in glaucoma.

A year later, it can be assumed that the undertaken measures have allowed to carry out 70% of the surgeries performed in the pre-pandemic period, even with reduced resources. The suspension of elective procedures, such as cataracts, has contributed to concentrate on emergency or high-priority surgeries, like glaucoma.

We suppose that the slowdowns in monitoring and treatment that occurred at the beginning of the pandemic partially led to the increase in cases of advanced and end-stage glaucoma and cyclo-photo ablative procedures. The closure of the operating rooms in the first quarter of the pandemic might have contributed to the worsening of the clinical conditions of some of the patients. The delay in the management of these patients could have determined irreversible functional visual damages.

From December 27th, 2020, as soon as the COVID-19 vaccination campaign started, for health professionals, the resources allocated to glaucoma surgery are slowly starting to be restored.

Glaucoma surgery deserves urgencies that cannot be overshadowed [14]. Considering the negative consequences that a delay in the management of glaucoma can have in terms of visual loss [15], we confide that our experience can be of some help in the organization of surgical assistance for other eye centers.

## Supplementary Information

Below is the link to the electronic supplementary material.

Supplementary file1 (DOCX 14 kb)

Supplementary file2 (DOCX 16 kb)

## Data Availability

Data are available, if requested.

## References

[1] (ISS) ISdS (2020) Epidemia Covid-19. Aggiornamento Nazionale 7 maggio 2020

[2] Sanità ISd (2020) Impatto dell’Epidemia Covid-19 sulla mortalità totale della popolazione residente periodo Gennaio-Novembre 2020. Istituto Superiore di Sanità e Istituto Nazionale di Statistica. https://www.iss.it/

[3] dell’Omo R, Filippelli M, Virgili G, Bandello F, Querques G, Lanzetta P, Avitabile T, Viola F, Reibaldi M, Semeraro F, Quaranta L, Rizzo S, Midena E, Campagna G, Costagliola C, Group EiIdC-pEs (2021) Effect of COVID-19-related lockdown on ophthalmic practice in Italy: a report from 39 institutional centers. Eur J Ophthalmol. 10.1177/11206721211002442 10.1177/11206721211002442 33724078

[4] Toro MD, Brézin AP, Burdon M, Cummings AB, Evren Kemer O, Malyugin BE, Prieto I, Teus MA, Tognetto D, Törnblom R, Posarelli C, Chorągiewicz T, Rejdak R (2021). Early impact of COVID-19 outbreak on eye care: insights from EUROCOVCAT group. Eur J Ophthalmol.

[5] Liebmann JM (2020). Ophthalmology and glaucoma practice in the COVID-19 era. J Glaucoma.

[6] Veritti D, Sarao V, Bandello F, Lanzetta P (2020). Infection control measures in ophthalmology during the COVID-19 outbreak: a narrative review from an early experience in Italy. Eur J Ophthalmol.

[7] Romano MR, Montericcio A, Montalbano C, Raimondi R, Allegrini D, Ricciardelli G, Angi M, Pagano L, Romano V (2020). Facing COVID-19 in ophthalmology department. Curr Eye Res.

[8] Sanità ISd (2021) Epidemia COVID-19, Aggiornamento Nazionale 3 Marzo 2021

[9] Brusini P, Filacorda S (2006). Enhanced glaucoma staging system (GSS 2) for classifying functional damage in glaucoma. J Glaucoma.

[10] Bissig A, Rivier D, Zaninetti M, Shaarawy T, Mermoud A, Roy S (2008). Ten years follow-up after deep sclerectomy with collagen implant. J Glaucoma.

[11] Ang GS, Varga Z, Shaarawy T (2010). Postoperative infection in penetrating versus non-penetrating glaucoma surgery. Br J Ophthalmol.

[12] Lachkar Y, Neverauskiene J, Jeanteur-Lunel MN, Gracies H, Berkani M, Ecoffet M, Kopel J, Kretz G, Lavat P, Lehrer M, Valtot F, Demailly P (2004). Nonpenetrating deep sclerectomy: a 6-year retrospective study. Eur J Ophthalmol.

[13] Quaranta L, Micheletti E, Riva I (2021). Response to letter to the editor: glaucoma surgery during the COVID-19 pandemic in Italy: how novel coronavirus has changed the surgical management of glaucoma patients. J Glaucoma.

[14] (AAO) AAoO (2020) List of ugernt and emergent ophthalmic procedures. https://www.aao.org/headline/list-of-urgent-emergent-ophthalmic-procedures. Accessed 10 Mar 2020

[15] Foot B, MacEwen C (2017). Surveillance of sight loss due to delay in ophthalmic treatment or review: frequency, cause and outcome. Eye (Lond).

