# Supplementary material for: Glaucoma surgery during the first year of the COVID-19 pandemic
Source: Int Ophthalmol. 2022 Apr 16;42(9):2881–7. doi: 10.1007/s10792-022-02278-6 (PMC9013211; doi:10.1007/s10792-022-02278-6)
Supplement: Supplementary file 1 — Supplementary file1 (DOCX 14 kb) [file 10792_2022_2278_MOESM1_ESM.docx]

Table 2 (Supplementary material). Regional origin of patients underwent glaucoma surgery during and before the COVID-19 pandemic.

|  | **Pre-Covid (%)** | **Covid (%)** | **p** |
| --- | --- | --- | --- |
| ***Regional Origin*** |  |  | 0.216 |
| **Veneto** | 53.2 | 59.9 |  |
| **Northern Italy** | 32.9 | 27.8 |  |
| **Mid-Italy** | 7.5 | 5.9 |  |
| **South and Islands** | 6.5 | 6.4 |  |
