# Supplementary material for: Glaucoma surgery during the first year of the COVID-19 pandemic
Source: Int Ophthalmol. 2022 Apr 16;42(9):2881–7. doi: 10.1007/s10792-022-02278-6 (PMC9013211; doi:10.1007/s10792-022-02278-6)
Supplement: Supplementary file 2 — Supplementary file2 (DOCX 16 kb) [file 10792_2022_2278_MOESM2_ESM.docx]

Table 3 (Supplementary material). Comparison of surgical procedures for Glaucoma in 2017, 2018 and 2019.

|  | 9^th^ March 2017-  8^th^ March 2018 | 9^th^ March 2018-  8^th^ March 2019 | 9^th^ March 2019-  8^th^ March 2020 | p |
| --- | --- | --- | --- | --- |
| ***Demographics*** |  |  |  |  |
| Surgical procedures, n | 651 | 664 | 701 | .184 |
| Patients, n | 411 | 447 | 494 | .096 |
| Males, n (%) | 204 (49.6) | 232 (51.9) | 258 (52.2) | .678 |
| Age, mean (SD) | 58.2  (24.0) | 57.4  (23.9) | 58.4  (23.4) | .902 |
| Advanced or End-Stage Glaucoma, n (%) | 21 (5.1) | 18 (4.0) | 20 (4.1) | .914 |
| Pediatric Glaucoma, n (%) | 73 (17.8) | 67 (15.0) | 69 (14.0) | .682 |
| General Anesthesia, n (%) | 222 (34.1) | 217 (32.7) | 227 (32.4) | .776 |
| ***Procedures, n (%)*** |  |  |  |  |
| Examination Under Anesthesia | 92 (14.1) | 88 (13.3) | 93 (13.3) | .867 |
| Trabeculotomy | 3 (0.5) | 5 (0.7) | 6 (0.9) | .666 |
| Trabeculectomy | 107 (16.4) | 114 (17.2) | 119 (17.0) | .935 |
| Deep Sclerectomy | 69 (10.5) | 63 (9.5) | 66 (9.4) | .719 |
| MIGS | 18 (2.8) | 20 (3.0) | 22 (3.1) | .920 |
| Baerveldt Tube Shunt | 80 (12.3) | 82 (12.3) | 87 (12.4) | .998 |
| Baerveldt revision | 72 (11.1) | 81 (12.2) | 90 (12.8) | .564 |
| Bleb revision | 45 (7.0) | 42 (6.3) | 47 (6.7) | .918 |
| Transcleral cyclophotocoagulation | 47 (7.2) | 42 (6.3) | 35 (5.0) | .236 |
| Phaco for narrow-angle or PEX syndrome | 105 (16.1) | 115 (17.4) | 121 (17.3) | 0.809 |
| Others* | 13 (2.0) | 12 (1.8) | 15 (2.1) | 0.907 |
